# Identification of a novel first-generation HIV-1 circulating recombinant form (CRF152_DG) among people living with HIV in Karachi, Pakistan

**DOI:** 10.1128/spectrum.00529-24

**Published:** 2024-05-21

**Authors:** Abdur Rashid, Li Kang, Feng Yi, Yimam Getaneh, Qingfei Chu, Sharaf Ali Shah, Syed Hani Abidi, Yiming Shao

**Affiliations:** 1 School of Medicine, Nankai University, Tianjin, China; 2 National Key Laboratory of Intelligent Tracking and Forecasting for Infectious Diseases, Chinese Center for Disease Control and Prevention, Beijing, China; 3 College of Life Sciences, Nankai University, Tianjin, China; 4 State Key Laboratory for Diagnosis and Treatment of Infectious Diseases, National Clinical Research Center for Infectious Diseases, National Medical Center for Infectious Diseases, Collaborative Innovation Center for Diagnosis and Treatment of Infectious Diseases, The First Affiliated Hospital, Zhejiang University School of Medicine, Hangzhou, China; 5 Ethiopian Public Health Institute, Addis Ababa, Ethiopia; 6 Bridge Consultants Foundation, Karachi, Pakistan; 7 Department of Biological and Biomedical Sciences, Aga Khan University, Karachi, Pakistan; 8 Department of Biomedical Sciences, School of Medicine, Nazarbayev University, Astana, Kazakhstan; 9 Changping Laboratory, Beijing, China; University of Miami, Miami, Florida, USA

**Keywords:** HIV-1 genetics, molecular epidemiology, phylogeny, genetic recombination, Pakistan, United Kingdom

## Abstract

The objective of this study was to characterize a novel circulating recombinant form of human immunodeficiency virus type 1 (HIV-1) among people living with HIV in Karachi, Pakistan. We conducted near-full-length genome (NFLG) sequencing on eight samples exhibiting D/G recombination signals in the *pol* gene region. We successfully obtained NFLG sequences (790–9,614; with reference to the HXB2 genome) from four of the eight samples and then conducted phylogenetic and recombination analyses on them. The four NFLG sequences from our study and one DG unique recombinant form previously identified in the United Kingdom (GenBank accession: MF109700) formed a distinct monophyletic cluster with an Shimodaira-Hasegawa approximate likelihood ratio test node support value of 100%. Bootscan analyses of the five NFLG sequences of DG recombinants showed that all five NFLGs shared the same unique mosaic pattern of recombination breakpoints between D and G clades, with two D fragments in the *pol* and *vif* regions inserted into a G backbone. Subregion phylogenetic analyses confirmed these sequences to be a novel circulating recombinant form (CRF) composed of subtypes D and G. The DG recombinant sequences were eventually designated as CRF152_DG by the Los Alamos HIV Sequence Database staff.

**IMPORTANCE:**

In Pakistan, the genetic diversity of human immunodeficiency virus type 1 (HIV-1) is becoming increasingly complex, compared to the early years of the epidemic that started after the detection of the first cases of HIV-1 in 1987 in Karachi. Based on the available molecular studies, two dominant HIV-1 clades, sub-subtype A1 and CRF02_AG, have been found to co-circulate with other clades, namely B, C, D, G, CRF01_AE, CRF35_A1D, and CRF56_cpx, in various urban areas of Pakistan. Several novel recombinant forms have also been detected. This first report of CRF152_DG highlights the complex nature of the HIV epidemic in Pakistan and emphasizes the importance of continual molecular surveillance (ideally based on whole-genome sequences) of HIV.

## INTRODUCTION

Human immunodeficiency virus type 1 (HIV-1) exhibits considerable genetic variability, which contributes to the emergence of viral variants. Recombination is a major mechanism that significantly contributes to the genetic diversity of HIV-1, driven by template switching during the replication cycle (1). Similarly, the co-circulation of distinct HIV-1 strains in a population increases the possibilities of coinfection, which may lead to the generation of novel recombinant forms (2, 3).

In the late 1980s, the first HIV-1 recombinant genome was reported (4). In the last two decades, different circulating recombinant forms (CRFs) and unique recombinant forms (URFs) have been identified worldwide. Among them, CRF01_AE, CRF02_AG, and CRF07_BC have become major epidemic strains in different countries (5). CRFs are recombinants of two or more subtypes that share the same pattern of recombination breakpoints along their near-full-length genome (NFLG) and have been identified in at least three epidemiologically unlinked individuals, while URFs are recombinants that do not meet the CRF criterion (6). Currently, 157 CRFs and numerous URFs have been identified (https://www.hiv.lanl.gov/), accounting for 22.8% of all HIV-1 infections globally (5).

Pakistan has reported a 78.5% increase in new HIV infections in the last decade (https://www.aidsdatahub.org). Currently, 210,000 people are registered in different HIV treatment centers across the country (7). In Pakistan, the HIV-1 genetic diversity is becoming increasingly complex, with sub-subtype A1 and CRF02_AG being the most predominant clades responsible for approximately 68.1% and 16.5% of HIV-1 infections (8, 9). However, other HIV-1 clades have been found in the country (8). Previous studies from our research group based on the HIV-1 *pol* region reported intersubtype recombinants CRF56_cpx and DG among HIV-1 infected individuals (10, 11), while near-full-length genome sequence analyses identified CRF02/A1 recombinants (12).

Karachi is the largest city in Pakistan, located on the coast of the Arabian Sea, with a population of over 17 million. According to the Sindh province HIV/AIDS epidemiology database, Karachi has the highest number of HIV-1 cases (*N* = 6,768) (13). The key HIV population groups in Karachi are people who inject drugs (PWID) and sex workers (14). Meanwhile, HIV-1 prevalence among men who have sex with men (MSM) has also increased (11%), making them another key HIV population group in Karachi (15). Previous studies have identified sub-subtype A1, CRF02_AG, CRF35_A1D, C, D, and G clades circulating among key HIV populations in Karachi (7, 16).

In the present study, we report the genetic characterization of novel DG recombinant strains, designated as CRF152_DG, exhibiting a mosaic structure identical to a previously identified DG URF in the United Kingdom (17). We propose that DG URFs previously identified in Pakistan (Karachi, Lahore, and Peshawar) (10, 18), the United Kingdom (17), Thailand (GenBank accession no: KF745710), China (19), Singapore (20), and India (21) are actually the CRF152_DG recombinant strains.

## MATERIALS AND METHODS

### Study samples

In our ongoing study on HIV phylogenetics in Karachi, we have identified a monophyletic transmission cluster consisting of eight DG recombinant sequences (based on partial *pol* sequences). To phylogenetically characterize these eight sequences, we selected 20 partial *pol* DG URF sequences previously identified in Pakistan (Karachi, Lahore, and Peshawar), the United Kingdom, Thailand, China, Singapore, India, Cyprus, Cameroon, and Kenya from the Los Alamos HIV Sequence Database (https://www.hiv.lanl.gov/) (Table S1). These sequences were subsequently used to construct a maximum likelihood (ML) phylogenetic tree using IQ-Tree v2.0 software (22). The ML tree showed that our eight DG recombinant sequences clustered with DG URF sequences from Pakistan, the United Kingdom, Thailand, China, Singapore, and India, which were designated as the “DG transmission cluster” (Fig. S1).

In the next step, we employed near-full-length genome amplification and sequencing to fully characterize the eight DG recombinant sequences. Written informed consent was obtained from all participants prior to blood collection. A unique laboratory identification number (MSM180, AB452, AKU-GT45, PWID108, DRM20, AKU-GT60, AF5, and AKU-GT) was given to each sample to ensure the confidentiality of the study participants. All experiments were conducted in accordance with approved guidelines and regulations.

### HIV-1 near-full-length genome amplification and sequencing

All eight samples were used to perform near-full-length genome sequencing following the methods described previously (23, 24). Briefly, the viral DNA was amplified into two overlapping fragments using a nested polymerase chain reaction with TaKaRa LA Taq polymerase (TaKaRa, Dalian, China), employing specific primers described in Table S2. Positive PCR amplicons were purified using the QIAquick Gel Extraction Kit (Qiagen, Germany) and sequenced on an ABI 3730XL sequencer using BigDye Terminators (Applied Biosystems, Foster City, CA, USA). Chromatograms were cleaned and assembled using Sequencher v5.4.6 (Gene Codes, Ann Arbor, MI, USA).

### Phylogenetic analysis

Of the eight samples, NFLGs were successfully sequenced from four samples. The four NFLGs were aligned with the HXB2 reference sequence using MAFFT v7 (25) and manually edited in BioEdit v7.2.5 software (26). Subsequently, the HIV BLAST tool, available at the Los Alamos HIV Sequence Database (https://www.hiv.lanl.gov/), was used to retrieve sequences showing high similarity to the four NFLGs. NFLG reference sequence alignment of HIV-1 M group subtypes and CRFs from the year 2020 were also retrieved from the Los Alamos HIV Sequence Database (https://www.hiv.lanl.gov/). The reference sequences and eight DG NFLGs (four from our study and one DG URF MF109700 from the United Kingdom that showed >93% similarity to our study DG recombinants, and randomly selected three DG URFs FJ623495, KP718916, and FJ388908 identified, respectively, in Kenya, Cameroon, and Cyprus), retrieved from the Los Alamos HIV Sequence Database, were aligned using MAFFT v7 (25) and manually edited using the BioEdit v7.2.5 software (26). The final NFLG alignment was used to generate a maximum likelihood phylogenetic tree using IQ-Tree v2.0 (22), with a general time-reversible plus gamma (GTR + G) model of nucleotide substitution, while the branch support was estimated using the Shimodaira-Hasegawa approximate likelihood ratio test (SH-aLRT), where SH-aLRT support value ≥90% was considered significant (27). The ML tree was visualized and edited in Figtree v1.4.4 (http://tree.bio.ed.ac.uk/software/figtree/).

### Recombination analysis

The Recombination Identification Program (RIP) (28) and jumping profile Hidden Markov Model (jpHMM) (29) programs available at the Los Alamos HIV Sequence Database (https://www.hiv.lanl.gov/) were used to detect recombination in the eight DG NFLG sequences. Furthermore, the recombination breakpoints in the eight DG NFLG sequences were analyzed using Simplot version 3.5.1 (30), which employs bootscanning and distance scanning approaches. Bootscanning was used to identify putative recombination breakpoints (31), and distance scanning was performed by computing the similarity between HIV clades using the Kimura 2-parameter model with a transition-transversion ratio of 2.0 (32). For this analysis, we used an alignment that included eight DG recombinant NFLG sequences, along with HIV-1 group M subtype reference sequences retrieved from the Los Alamos HIV Sequence Database (https://www.hiv.lanl.gov/).

Furthermore, to confirm the inter-subtype recombination breakpoints, ML phylogenetic trees were constructed using IQTree v2.0 software (22). For this analysis, alignment files comprising each segment of the recombinant genomes, along with HIV-1 group M subtype reference sequences, were used. The ML trees were constructed using the GTR + G model of nucleotide substitution, while the branch support was estimated using the Shimodaira-Hasegawa approximate likelihood ratio test, where SH-aLRT support value ≥ 90% was considered significant (27). All the sub-genomic phylogenies were visualized in Figtree v1.4.4 (http://tree.bio.ed.ac.uk/software/figtree/). Finally, the genomic structure of the DG recombinant was generated with the Recombinant HIV-1 Drawing Tool available at the Los Alamos HIV Sequence Database (https://www.hiv.lanl.gov/).

### HIV-1 drug resistance and co-receptor tropism analysis

The four individual *pol* sequences, from our study, were screened for drug resistance mutations (DRMs) in the protease, reverse transcriptase, and integrase regions using the Stanford HIV Drug Resistance Database program (https://hivdb.stanford.edu/) and confirmed using the International AIDS Society 2022 update of the drug resistance mutations in HIV-1 (33). HIV-1 co-receptor usage was determined using the Geno2pheno v3.4 tool (https://www.geno2pheno.org). A threshold for the false-positive rate (FPR) was set at 2% to delineate viral phenotypes. FPR values ≤ 2% were designated as CXCR4 tropic or CCR5/CXCR4 (R5X4) dual tropic, while FPR values > 2% were designated as CCR5 tropic variants (34, 35).

## RESULTS

### Sample characteristics

All eight individuals included in this study belong to different districts of Karachi. Their HIV infection was diagnosed in 2008 (*n* = 1), 2015 (*n* = 1), 2018 (*n* = 1), 2020 (*n* = 1), and 2021 (*n* = 3), while one individual had an unknown year of diagnosis. The median age of the eight individuals was 27 years (interquartile range: 20). Seven were male, and the gender of one individual was not recorded. Injecting drugs was the risk behavior for two individuals, unprotected homosexual contact for one, and unknown for five.

### Phylogenetic analysis of NFLG sequences

Out of eight samples, NFLGs were successfully amplified and sequenced from only four samples. The NFLG sequences derived from the four subjects, coded as AKU-GT45, AKU-GT60, DRM20, and AF5, were, respectively, 8,821, 8,834, 8,845, and 8,833 base pairs in size spanning from 5′ gag region to 3′ LTR region, corresponding to HIV-1 HXB2 strain nucleotide position 790–9,614.

**Fig 1:**
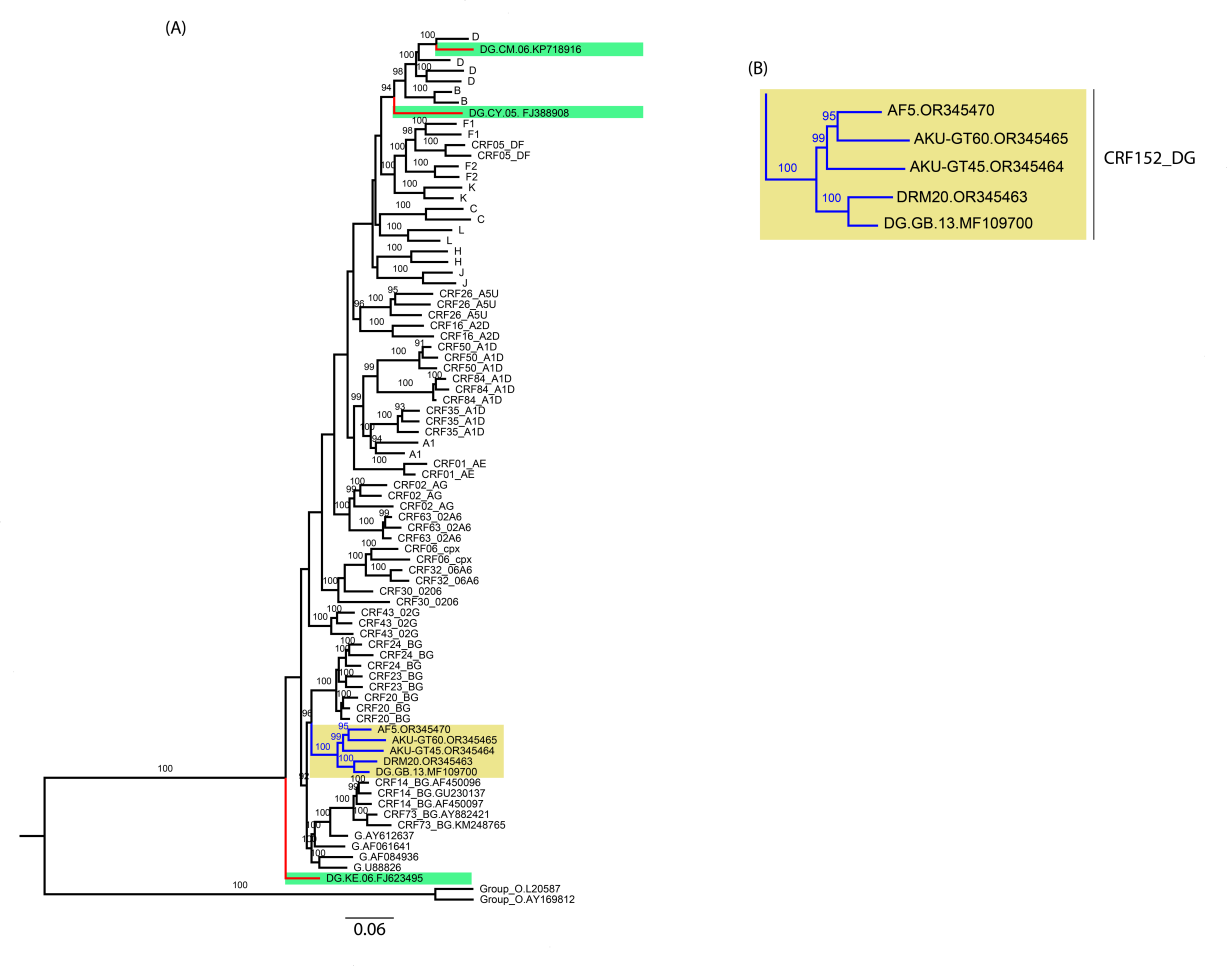
Phylogenetic analysis based on near-full-length genome sequences of DG recombinant. (**A**) A maximum likelihood phylogenetic tree was constructed using HIV-1 subtype and CRF reference sequences retrieved from the Los Alamos HIV Sequence Database (https://www.hiv.lanl.gov/). Five CRF152_DG NFLG sequences [four from our study and one DG from the United Kingdom (GenBank accession: MF109700)] are colored blue and highlighted with dark yellow, while three DG URFs retrieved from the Los Alamos HIV Sequence Database are colored red and highlighted with light green. The HIV-1 reference sequences are colored black. (**B**) The enlarged CRF152_DG cluster. Only SH-aLRT node support values ≥ 90% are shown.

The ML phylogenetic tree was constructed using the NFLG sequences from our study and four DG NFLG sequences retrieved from the HIV LANL database, along with HIV-1 subtype and CRF reference sequences. Phylogenetic analysis showed that our study sequences (AKU-GT60, AF5, AKU-GT45, and DRM20) clustered with a DG URF identified in the United Kingdom (accession no.: MF109700) and formed a monophyletic cluster with a SH-aLRT node support value of 100% that was distinct from other known HIV-1 subtypes and CRFs, indicating a potentially new CRF circulating in Pakistan, which was later designated as CRF152_DG by the Los Alamos HIV Sequence Database staff (Fig. 1A).

### Recombination analysis of NFLG sequences

**Fig 2:**
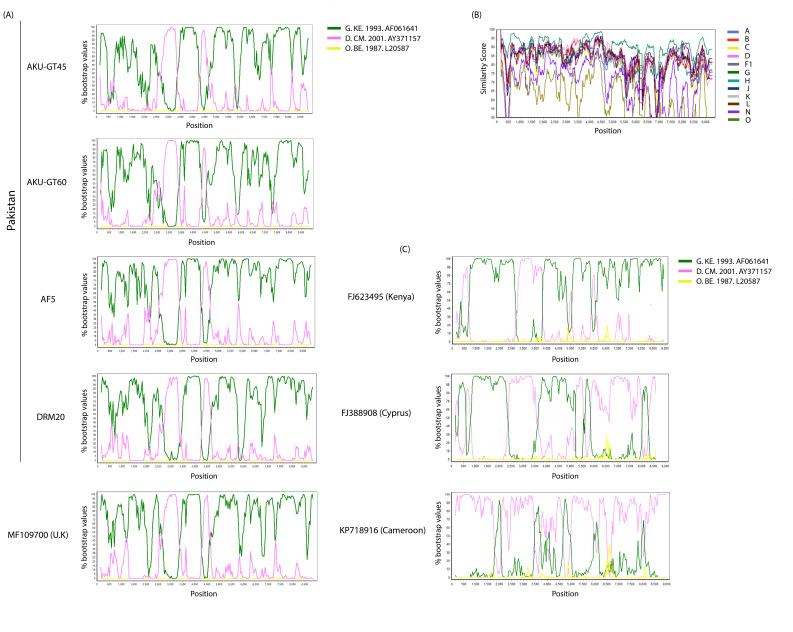
Recombination analyses of the eight DG NFLGs sequences. (**A**) Bootscan analysis of the five CRF152_DG NFLG sequences (four DG recombinants from our study and one DG URF from the United Kingdom). (**B**) Similarity plot analysis of the same five CRF152_DG NFLG sequences. (**C**) Bootscan analysis of the three NFLGs DG URFs retrieved from the Los Alamos HIV Sequence Database (https://www.hiv.lanl.gov/). Similarity and bootscan analyses were conducted using a window size of 300 bp and a step size of 40 bp along with HIV references of subtypes D and G, and representative reference sequences of HIV-1.

**Fig 3:**
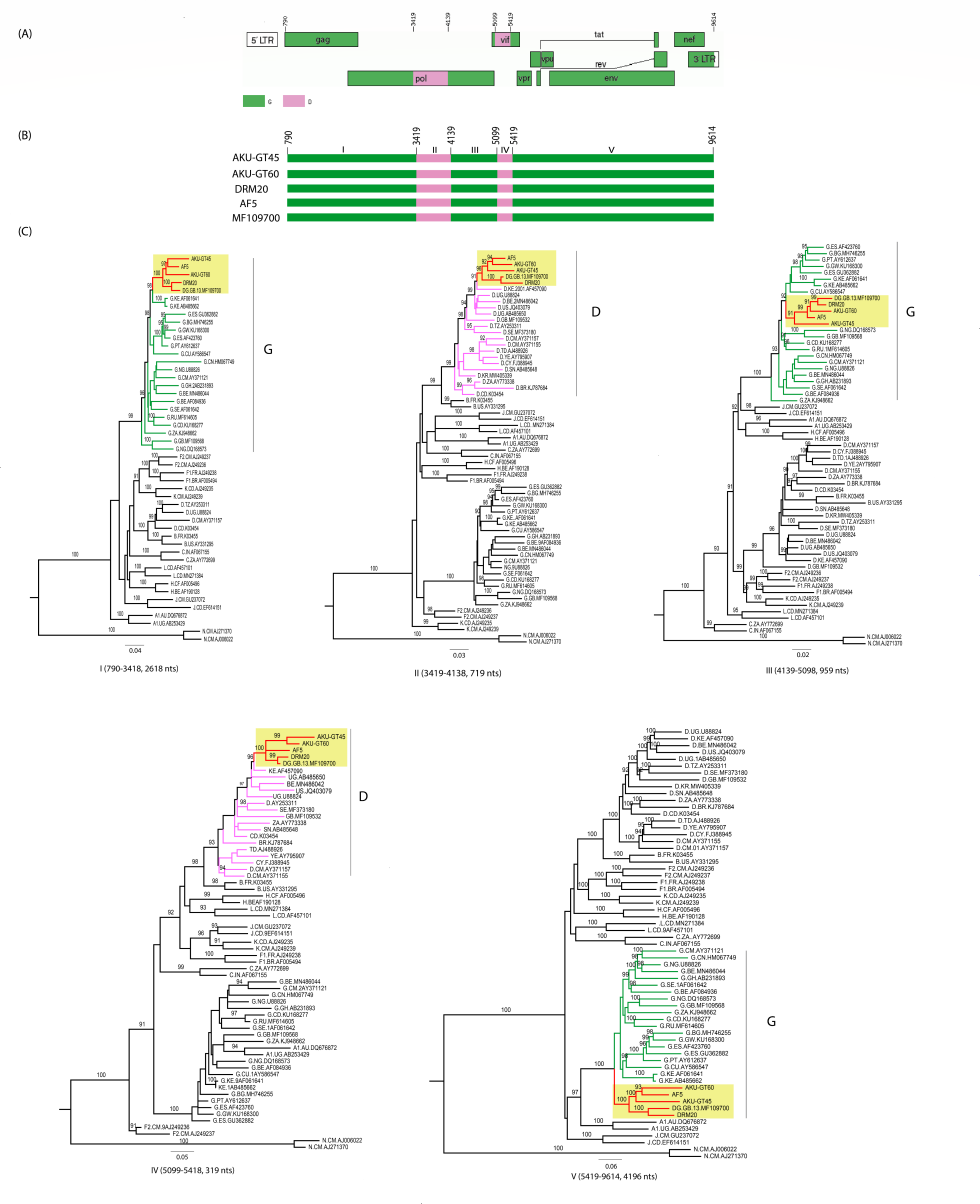
Recombination breakpoints and subregion phylogenetic analyses of CRF152_DG. (**A**) The genome structure of CRF152_DG generated using the Recombinant HIV-1 Drawing Tool. The D and G fragments within the CRF152_DG NFLG are shown in lavender and green color, respectively. (**B**) Genomic structures of the five CRF152_DG NFLG sequences. (**C**) ML phylogenetic trees of the five clade D and G fragments identified by bootscan and similarity plot analyses. CRF152_DG sequences are colored red with a gold background, while D, G, and other HIV-1 subtype reference sequences in each ML tree are colored lavender, green, and black. Nucleotide positions of each subtype fragment in the CRF152_DG NFLG were numbered according to HIV-1 reference strain HXB2 (K03455). Only SH-aLRT node support values ≥ 90% are shown.

The RIP and jpHMM analyses of the five DG NFLG sequences confirmed the recombination between clades D and G (Fig. S2 and S3). Similarly, the bootscan and similarity plot analyses indicated that the four NFLG sequences showed the same pattern of recombination and shared four recombination breakpoints (Fig. 2A and B) with the DG URF from the United Kingdom (17). Interestingly, the four NFLG sequences did not share the recombination patterns with the three DG URFs that did not cluster with our study sequences (Fig. 2C). Bootscan and similarity plot analyses also identified four unique inter-subtype recombination breakpoints that were identical in all five NFLGs (four from our study and one from the United Kingdom), which divided the recombinant genome into five segments. The four unique inter-subtype recombination breakpoints between clades D and G were located at nucleotide positions 3,419 (I; *pol*), 4,139 (II; *pol*), 5,099 (III; *vif*), and 5,419 (IV; *vif*) with reference to the HXB2 genome (Fig. 3A). The genomic structure of the DG recombinants comprised 88.2% of clade G and 11.8% of the two fragments of clade D, which spanned from nucleotide positions 3,419–4,138 (HXB2) and 5,099–5,418 (HXB2), covering a portion of the *pol* and *vif* regions, respectively (Fig. 3A and B).

The mosaic structure of the five DG recombinants (four from our study and one from the United Kingdom) was further confirmed by ML phylogenetic analyses, showing regions I (HXB2: 790–3,418 nts), III (HXB2: 4,139–5,098 nts), and V (HXB2: 5,419–9,614 nts) to cluster with clade G reference sequences, while regions II (HXB2: 3,419–4,138 nts) and IV (HXB2: 5,099–5,418 nts) to cluster with clade D reference sequences (Fig. 3C).

### HIV-1 drug resistance and co-receptor tropism analysis

Among the four study participants, only one individual was antiretroviral therapy (ART)-experienced (DRM20), while the ART history of the three individuals was unknown. HIV drug resistance was detected in three individuals: two individuals (DRM20 and AKU-GT60) showed resistance to non-nucleoside reverse transcriptase inhibitors (NNRTIs), while one individual (AKU-GT45) showed resistance to nucleoside reverse transcrptase inhibitors (NRTI).

The most common DRMs were M184V, associated with resistance against NRTIs, and K103N, associated with resistance against NNRTIs, detected in the same two individuals. Similarly, DRMs K65R and Y115F, associated with resistance against NRTIs, and V179E, Y181C, and H221Y, associated with resistance against NNRTIs, were only observed in one individual. No mutation was detected in the protease region, while mutation E92A was observed in the integrase region of HIV sequenced from one individual.

HIV-1 co-receptor tropism analysis, performed using the envelope gp120 V3 loop amino acid sequence, revealed that the four DG recombinants were CCR5 (R5) usage variants, exhibiting FPR values of 5.3%, 87.4%, 17.6%, and 76.8%, respectively. Of the four DG recombinants, two sequences (AKU-GT45 and DRM20) had a V3 loop crown motif comprising GPGQ, whereas two sequences (AKU-GT60 and AF5) had a V3 loop crown motif comprising GPGH.

## DISCUSSION

A molecular surveillance study was conducted in 2022 to address HIV-1 genetic diversity, drug resistance, and transmission linkages among HIV-1-infected key populations in Karachi, Pakistan. Individuals participating in this study were found to be infected by sub-subtype A1 (40%), CRF02_AG (33.2%), subtype C (7.8%), subtype D (2.2%), and subtype G (1.86%) (A. Rashid and L. Kang, et al., unpublished data). These results are consistent with the prevalent strains observed among people living with HIV (PLHIV) in Pakistan (13, 36). Consequently, a DG transmission cluster, comprising eight sequences, was detected among PLHIV in Karachi. In this study, we performed near-full-length genome sequencing to characterize the recombination patterns of those suspected DG recombinant forms.

Out of eight, four DG recombinant NFLG sequences were successfully sequenced. Bootscan and similarity plot analyses showed that the DG recombinants shared the same pattern of recombination along their genome, with four breakpoints located in the *pol* and *vif* regions (Fig. 2A and B).

Phylogenetic analysis showed that the four DG NFLGs recombinant clustered with a DG URF from the United Kingdom (GenBank accession: MF109700) and exhibited high sequence similarity, exceeding 93%, with MF109700. This finding is further supported by our previous study (10), which identified a DG URF in Karachi closely related to the DG URF detected in the United Kingdom (17). However, no information exists about the introduction of subtypes D and G in Pakistan, even though the prevalence of subtypes D and G is gradually increasing (13, 36), and new recombinant strains have frequently been reported in the country (11, 12, 18).

The global distribution of HIV-1 subtypes is predominantly driven by socioeconomic shifts and immigration, while the regional expansion of specific variants is often attributed to the unique features of the virus and the opportunity for transmission through specific routes (37). Approximately 6 million Pakistanis are living abroad, particularly in the Gulf States, Europe, and the United Kingdom (38). It is possible that the migration of infected individuals, especially those with unknown or undisclosed HIV status, and their subsequent engagement in high-risk behaviors may have introduced newer variants in the country (39, 40). The detection of new recombinant forms in Karachi suggests an increase in the genetic complexity of the HIV-1 epidemic in Pakistan. Karachi, being the business hub of the country, receives an estimated 45,000 migrants, including refugees, workers, and repatriating Pakistanis, every month (41). Since the importation of the first HIV cases by repatriated Pakistanis from the Gulf States in 1987 in Karachi, sub-subtype A1 has become the most prevalent strain, accounting for 68.1% of HIV infections, and is frequently detected among key populations, including PWID and MSM (8, 42). Similarly, phylogenetic analysis of Pakistani HIV-1 sequences showed linkages with strains from neighboring countries, including Afghanistan and India (43).

Subtype D is the seventh most prevalent HIV-1 clade, accounting for 2.7% of all global infections, with the highest prevalence in the Middle East and North Africa at 44.4% and the Caribbean at 26% (5). Additionally, subtype D is associated with a higher viral load, a faster decline in CD4 (+) T-cell counts, and increased production of cytokines compared to subtype A (44, 45). Similarly, subtype G has a complex relationship with CRF02_AG; for example, approximately 80% of individuals infected with recombinant strains in Cameroon have a genome segment of subtype G (46). Intersubtype recombination between D and G subtypes has been reported among MSM and heterosexual populations in Kenya and Cyprus (47, 48). As per the current HIV nomenclature rules, the assignment of a new CRF requires two NFLG sequences in conjunction with partial sequences of a third strain (6). Our results suggest that the DG transmission cluster belongs to a new circulating recombinant form and is designated as CRF152_DG.

Drug resistance mutation analysis of the four CRF152_DG sequences showed the presence of DRMs K65R, Y115F, and M184V associated with resistance against NRTIs. DRMs K103N, V179E, Y181C, and H221Y were associated with resistance against NNRTIs. The DRM E92A, albeit a minor mutation, was associated with resistance against integrase inhibitors (being reported for the first time in a Pakistani HIV sequence). This indicates the importance of integrase inhibitor genotyping resistance testing. Co-receptor tropism analysis revealed that all four CRF152_DG strains exhibited CCR5 (R5) usage variants, with distinct V3 loop crown motifs (GPGQ and GPGH).

### Conclusion

This study reports a novel HIV-1 circulating recombinant form comprising subtypes D and G and designated as CRF152_DG. The emergence of novel HIV-1 recombinant forms in Pakistan suggests an increase in the genetic diversity of HIV in the country. Further molecular epidemiological surveillance studies can suggest whether this strain, especially the resistant strain, is transmitting to other areas of Pakistan.

## Data Availability

The sequences were deposited to the GenBank and accession number (s) can be found below: https://www.ncbi.nlm.nih.gov/genbank/, OR345466, OR345467, OR345468, OR345469 for the partial pol region and OR345463, OR345464, OR345465, and OR345470 for the near-full-length genomes.

## SUPPLEMENTAL MATERIAL

Supplemental material spectrum.00529-24-s0001.docx Tables S1 and S2; Fig. S1 to S3.

ASM does not own the copyrights to Supplemental Material that may be linked to, or accessed through, an article. The authors have granted ASM a non-exclusive, world-wide license to publish the Supplemental Material files. Please contact the corresponding author directly for reuse.
