# Supplementary material for: Identification of a novel first-generation HIV-1 circulating recombinant form (CRF152_DG) among people living with HIV in Karachi, Pakistan
Source: Microbiol Spectr. 2024 May 21;12(7):e00529-24. doi: 10.1128/spectrum.00529-24 (PMC11218485; doi:10.1128/spectrum.00529-24)
Supplement: Supplemental material — Tables S1 and S2; Fig. S1 to S3. [file spectrum.00529-24-s0001.docx]

**Supplementary Table S1: DG URF partial *pol* sequences used for ML phylogenetic analysis**

| **Accession no** | **Subtype (URF)** | **Country** | **Sampling Year** |
| --- | --- | --- | --- |
| MT223598 | DG | Pakistan (Karachi) | 2015 |
| MT223600 | DG | Pakistan (Karachi) | 2019 |
| OP918925 | DG | Pakistan | 2022 |
| MT223599 | DG | Pakistan (Karachi) | 2019 |
| MT176096 | DG | Pakistan (Peshawar) | 2014 |
| MT223602 | DG | Pakistan (Karachi) | 2019 |
| MN336504 | DG | Pakistan (Lahore) | 2017 |
| MK927054 | DG | Pakistan (Karachi) | 2017 |
| MN410953 | DG | Pakistan (Lahore) | 2017 |
| MN410952 | DG | Pakistan (Lahore) | 2017 |
| MN410954 | DG | Pakistan (Lahore) | 2017 |
| MN336505 | DG | Pakistan (Lahore) | 2017 |
| KF745710 | DG | Thailand (Bangkok) | 2013 |
| EU715229 | DG | Singapore | 2005 |
| MF109700 | DG | United Kingdom (London) | 2013 |
| KX583227 | DG | India | 2013 |
| MN797254 | DG | China | 2010 |
| FJ623495 | DG | Kenya | 2006 |
| FJ388908 | DG | Cyprus | 2005 |
| KP718916 | DG | Cameroon | 2006 |

URF; unique recombinant form, ML; maximum-likelihood

**Supplementary Table S2: Primers used in this study for nested PCR**

| Fragment | Position | Primer name | Sequence | Direction |
| --- | --- | --- | --- | --- |
| **1^st^** | 0538-0571 | F1.5 | CCTTGAGTGCTTCAAGTAGTGTGTGCCCGTCTGT | Forward |
|  | 5219-5193 | F1.3 | CCTARTGGGATGTGTACTTCTGAACTT | Reverse |
| **2^nd^** | 0634-0650 | F2.5 | AGTGGCGCCCGAACAGG | Forward |
|  | 5066-5041 | F2.3 | ATCATCACCTGCCATCTGTTTTCCAT | Reverse |
|  |  |  |  |  |
| **1^st^** | 4883-4912 | L1.5 | 5-AAAAATTCAAAATTTTCGGGTTTATTACAG-3 | Forward |
|  | 9607-9636 | L1.3 | TGAAGCACTCAAGGCAAGCTTTATTGAGGC | Reverse |
|  | 9611-9642 | LOW | ACTACTTGAAGCACTCAAGGCAAGCTTTATTG | Reverse |
| **2^nd^** | 4900-4923 | L2.5 | 5-GGGTTTATTACAGRGACAGCAGAG-3 | Forward |
|  | 9591-9612 | L2.3 | TGAGGCTTAAGCAGTGGGTTCC | Reverse |

Primers F1.5, F1.3 (up primers), and L1.5, L1.3, and LOW (down primers) were used for the first round of PCR, while primers F2.5, F2.3 (up primers), and L2.5, L2.3 (down primers) were used for the second round of PCR.

**
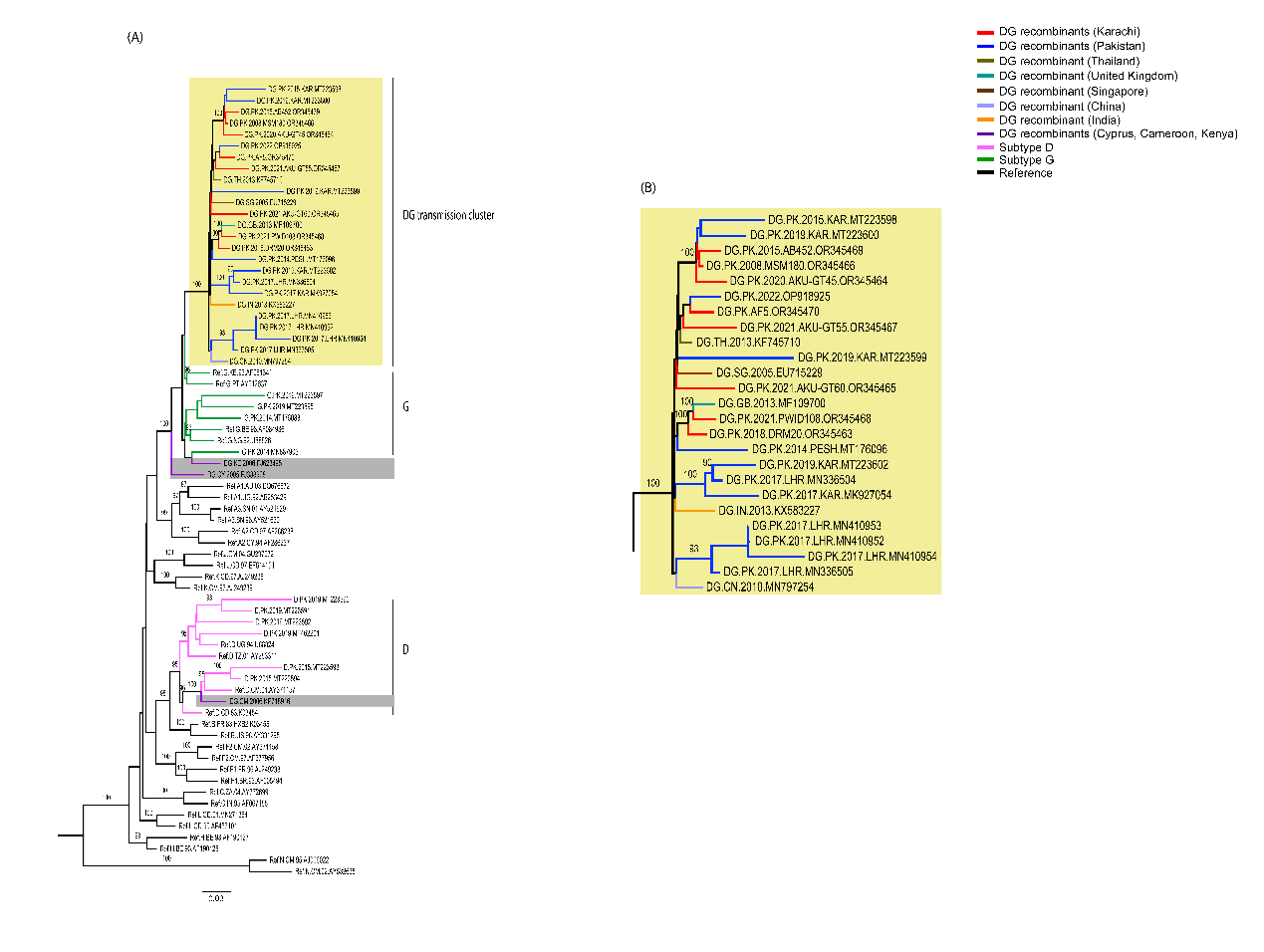
**

**FIG S1** Maximum likelihood phylogenetic tree of the DG transmission cluster based on the HIV-1 partial pol region (HXB2 2253-3315). (A) The DG recombinant sequences, amplified from PLHIV from Karachi, Pakistan, are colored red. Only SH-aLRT node support values ≥90% are shown. DG URF sequences retrieved from the Los Alamos HIV Sequence Database (https://www.hiv.lanl.gov/) are colored blue (Pakistan), dark teal (UK), dark green (Thailand), brown (Singapore), gold (India), light blue (China), and DG URF identified in Cyprus, Kenya, and Cameroon are colored purple and highlighted light grey, while HIV-1 subtype D, G, and other HIV-1 subtypes reference sequences are colored lavender, green, and black. (B) DG transmission cluster. The scale indicates nucleotide substitution/site (0.03).


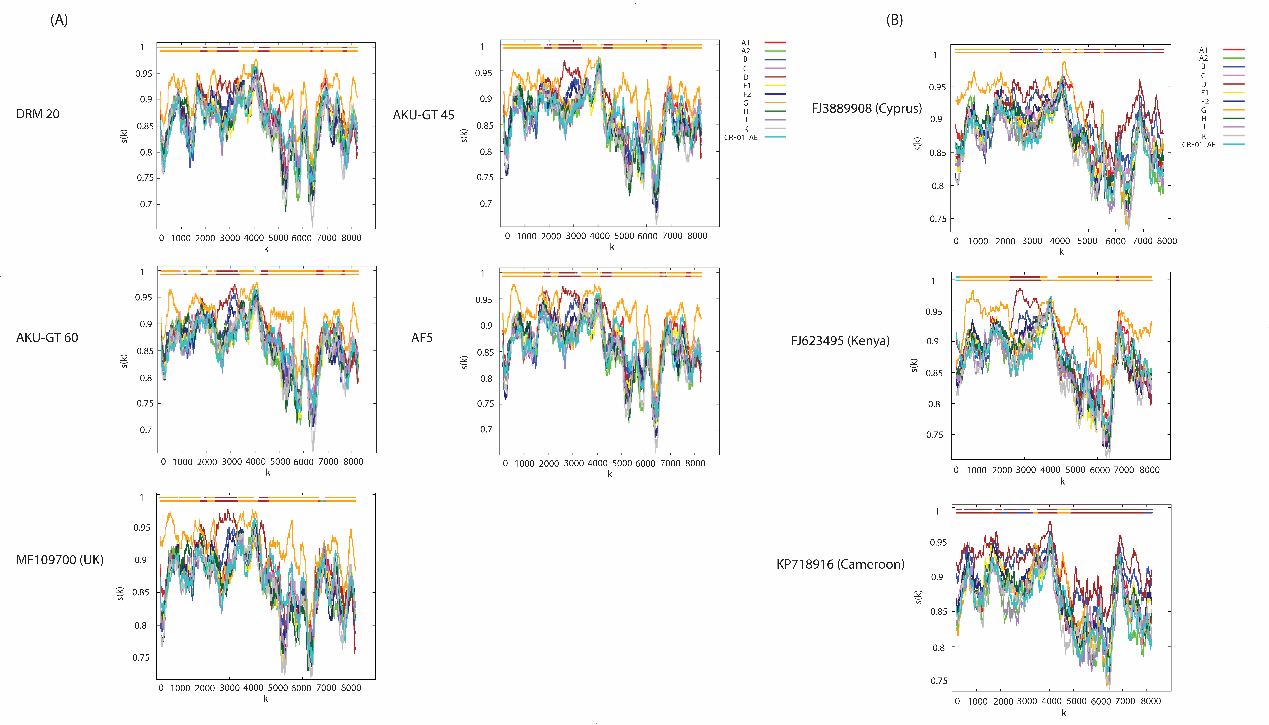


**FIG S2** Recombination pattern observed in RIP analysis, with a window size of 400 and a 90% confidence threshold. (A) Recombination pattern of five CRF152_DG NFLG sequences (four DG recombinants from our study and one DG URF from the United Kingdom). (B) Recombination pattern of the three NFLGs DG URFs retrieved from the Los Alamos HIV Sequence Database (<https://www.hiv.lanl.gov/>).


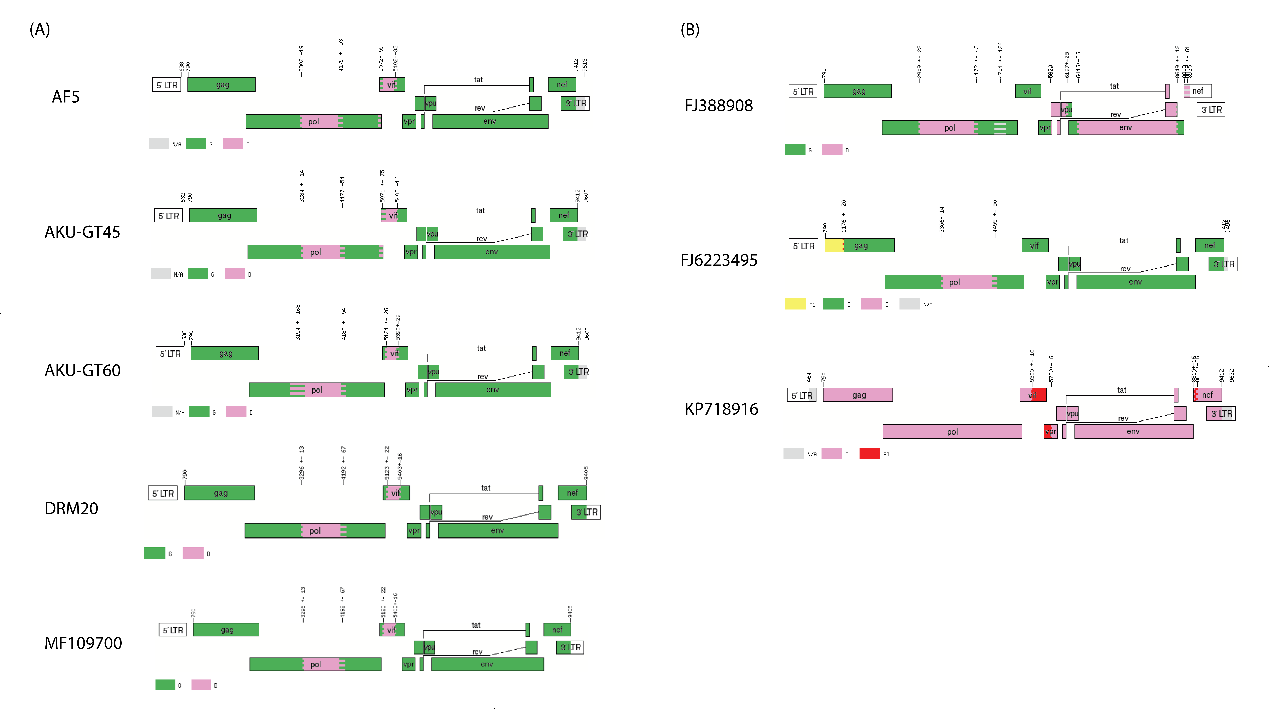


**FIG S3** Recombination pattern observed in jpHMM analysis (A) Recombination pattern of five CRF152_DG NFLG sequences (four DG recombinants from our study and one DG URF from the United Kingdom). (B) Recombination pattern of the three NFLGs DG URFs retrieved from the Los Alamos HIV Sequence Database (<https://www.hiv.lanl.gov/>).
